# Nuclear and *Wolbachia*-based multimarker approach for the rapid and accurate identification of tsetse species

**DOI:** 10.1186/s12866-018-1295-4

**Published:** 2018-11-23

**Authors:** Antonios A. Augustinos, Irene K. Meki, Guler Demirbas-Uzel, Gisele M. S. Ouédraogo, Aggeliki Saridaki, George Tsiamis, Andrew G. Parker, Adly M. M. Abd-Alla, Kostas Bourtzis

**Affiliations:** 1 Insect Pest Control Laboratory, Joint FAO/IAEA Division of Nuclear Techniques in Food and Agriculture, Vienna International Centre, P.O. Box 100, 1400 Vienna, Austria; 2 Ecole National de l’Elevage et de la Santé Animale, 03 BP 7026, Ouagadougou 03, Burkina Faso; 3 Department of Environmental and Natural Resources Management, University of Patras, Agrinio, Greece

**Keywords:** *Glossina*, Sterile insect technique, Internal transcribed spacer 1 (ITS1), Integrative taxonomy, Symbiosis

## Abstract

**Background:**

Tsetse flies (Diptera: Glossinidae) are solely responsible for the transmission of African trypanosomes, causative agents of sleeping sickness in humans and nagana in livestock. Due to the lack of efficient vaccines and the emergence of drug resistance, vector control approaches such as the sterile insect technique (SIT), remain the most effective way to control disease. SIT is a species-specific approach and therefore requires accurate identification of natural pest populations at the species level. However, the presence of morphologically similar species (species complexes and sub-species) in tsetse flies challenges the successful implementation of SIT-based population control.

**Results:**

In this study, we evaluate different molecular tools that can be applied for the delimitation of different *Glossina* species using tsetse samples derived from laboratory colonies, natural populations and museum specimens. The use of mitochondrial markers, nuclear markers (including internal transcribed spacer 1 (ITS1) and different microsatellites), and bacterial symbiotic markers (*Wolbachia* infection status) in combination with relatively inexpensive techniques such as PCR, agarose gel electrophoresis, and to some extent sequencing provided a rapid, cost effective, and accurate identification of several tsetse species.

**Conclusions:**

The effectiveness of SIT benefits from the fine resolution of species limits in nature. The present study supports the quick identification of large samples using simple and cost effective universalized protocols, which can be easily applied by countries/laboratories with limited resources and expertise.

**Electronic supplementary material:**

The online version of this article (10.1186/s12866-018-1295-4) contains supplementary material, which is available to authorized users.

## Background

Tsetse flies are responsible for the cyclic transmission of trypanosomes, causative agents of sleeping sickness or human African trypanosomosis (HAT) in humans and nagana or animal African trypanosomosis (AAT) in livestock [1, 2]. There are about 31 tsetse fly species and sub-species in *Glossina* genus (Diptera: Glossinidae), distributed in 37 sub-Saharan African countries. However, only 8-10 of these species are of economic importance [3].

Due to the lack of vaccines against trypanosomes and increasing resistance of the AAT parasites to available drugs [4, 5], vector control remains the most effective way of managing African trypanosomosis [6]. Some of the vector control strategies that have been applied for the control of trypanosomosis include the use of sequential aerosol technique (SAT) [7], stationery attractive devices, live bait technique and sterile insect technique (SIT) [8–10]. The SIT involves production of large numbers of the target insect species in specialized rearing facilities followed by sterilization of the males by irradiation [11]. The sustained and systematic release of the sterile males over the target area in large numbers out-competes the wild male population for mating with wild females. Mating of mass -produced sterile males with wild females leads to no offspring and subsequent decrease of the targeted population [12]. SIT is a species-specific and environmental friendly control method that has been successfully applied for the eradication of a population of *Glossina austeni* from Unguja Island in Zanzibar [13].

The correct species identification is of crucial importance for successful SIT applications. Several methods have been applied to identify tsetse species, including morphological characters such as external genitalia of males, their habitat requirements and host preference [10]. Based on these characters, the *Glossina* species are divided into three distinct taxonomic groups: *morsitans*, *palpalis* and *fusca* [14]. However, delimitation of closely related species and/or subspecies remains challenging.

In addition to morphological taxonomic identification of *Glossina* species, molecular and genetic markers have also been used in the last decades. Nuclear markers, such as ITS1 and ITS2, were reported to distinguish some of the species based on the size and/or specificity of the amplicons, as revealed by both agarose gel electrophoresis and sequencing [15–18]. Microsatellite markers have also been developed for different *Glossina* species and have provided encouraging results regarding their potential use in phylogenetic analysis and species identification [19–23]. Mitochondrial markers, including cytochrome oxidase 1 (COI), cytochrome oxidase 2 (COII), cytochrome b (CYTB), 16S rRNA, and NADH dehydrogenase 2 (ND2), have also been implemented for the phylogenetic analysis of *Glossina* species, based on DNA sequencing [15–17, 24–27]. The availability of polytene chromosomes in *Glossina* and the development of polytene chromosome maps provide additional genetic tools that can shed light on specific chromosomal banding pattern changes and / or rearrangements that could provide diagnostic characters for species identification [28–31].

A previously neglected parameter regarding speciation is the development of intimate relationships of the tsetse fly with bacterial symbionts, such as *Wigglesworthia glossinidia*, *Sodalis glossinidius,* and *Wolbachia*, that may alter the host’s behavior [32–35]. *Wolbachia* is obligatory intracellular and maternally transmitted and is known to cause reproductive alterations and cytoplasmic incompatibility (CI) [36]. CI is mainly expressed as embryonic mortality when an infected male mates with an uninfected female (unidirectional CI) [37] or when the male and female crossed harbor different and mutually incompatible *Wolbachia* strains (bidirectional CI) [38]. Such incompatibilities lead to restriction of gene flow among natural populations and can be both ‘accelerators’ and diagnostic markers of speciation [39]. Another aspect of symbiosis that could be exploited is the presence of ancient, species-specific, horizontal gene transfer events in the host’s chromosomal DNA. Such events have been demonstrated in *Glossina*, through the presence of fixed chromosomal introgressions of *Wolbachia* (only in *Glossina morsitans morsitans* up to now) and can provide additional diagnostic markers [40, 41].

Regarding the delimitation of closely related species and given that speciation can be driven through different or combined forces, integrative taxonomy suggests the utilization of multidisciplinary approaches for the inference of robust conclusions regarding species limits and phylogenetic relationships [42–46]. The utilization of a single marker, or a single class of tightly linked markers (such as mitochondrial genes), although easy to universally apply, is not expected to provide beyond doubt species identification [47, 48]. The fact that the phylogenetic signal of mitochondrial markers can be masked or altered by the presence of reproductive symbionts, such as *Wolbachia* (through, for example, mitochondrial sweeps) and the limitation that mitochondrial markers are unable to identify hybrids among closely related species (important in hybridizing zones of closely related species) also points to the need for ‘the more, the better’ approaches in species delimitation [49]. Previous studies also in tsetse flies have documented that different classes of markers may provide either a differential depth of analysis or even contradicting results [15, 17, 50].

Besides robustness, it is critical to develop diagnostic tools that can be applied quickly, easily, massively and cost effectively. This can be done by integrating different classes of markers and by utilizing different resolution techniques, such as gel electrophoresis and sequencing. Such integrated approaches allow the screening of many samples and many individuals per sample with reduced cost in a relatively short time and without the need of highly specialized equipment/skills.

Here we report the evaluation of different classes of molecular markers (nuclear ITS1, nuclear microsatellites, mitochondrial genes, and the *Wolbachia* infection status) for the identification of tsetse taxa. We evaluated these tools against tsetse laboratory colonies that were used as reference material. At the same time, we correlated our data with previously published sequences and data from tsetse museum specimens. Finally, we evaluated the discriminative power of ITS 1 amplicon electrophoresis through the genotyping of an extended collection of samples derived from nature. The main goal of this study was to develop a set of markers and analytical approaches that can quickly and cost effectively support the morphometric taxonomy or even stand alone to identify *Glossina* species.

## Methods

### Material used

#### Laboratory colonies

*Glossina* species maintained at the Insect Pest control Laboratory (IPCL) of the Joint FAO/IAEA Programme of Nuclear Applications in Food and Agriculture (NAFA) were used in this analysis. The species were *Glossina pallidipes, G. morsitans morsitans, G. morsitans centralis, G. palpalis gambiensis, G. fuscipes fuscipes,* and *G. brevipalpis*. Identification of the fly samples to species was based on standard morphological characters [14]. As morphological characters are not reliable for subspecific identification, the subspecific laboratory colonies were assigned based on the conventional designation for the place of origin. Details of the *Glossina* species and colonies used in this study are provided in Table 1. All the tsetse colonies are fed on heated, defibrinated bovine blood for 10-15 min, three days per week using an *in vitro* membrane feeding technique [51].

**Table 1:** *Glossina* samples analyzed in this study

*Glossina* species	Collection site	Original collection date	Details	Origin ^a^	*N*
*G. pallidipes*	Uganda (Tororo)	1975	1978 IPCL (from Institute of Experimental Entomology, Amsterdam, The Netherlands)	L	8
	Ethiopia (Arba Minch)	1997-2001	2005 IPCL (Arba Minch colony)	L	8
*G. m. morsitans*	Zimbabwe	1968	1972 IPCL (from Bristol laboratory colony)	L	8
*G. m. centralis*	Tanzania	N/A	1999 IPCL	L	8
*G. p. gambiensis*	Burkina Faso	1972	2005 IPCL (from CIRDES laboratory colony)	L	8
	Senegal (Pout)	2009	2009 IPCL	L	8
*G. f. fuscipes*	Central Africa Republic	1986	2009 IPCL	L	8
*G. brevipalpis*	Kenya (Shimba hills)	1987	2002 IPCL	L	8
*G. tachinoides*	Burkina Faso	N/A	CIRDES	L	12
*G. m. submorsitans*	Burkina Faso	N/A	CIRDES	L	12
total					88
*G. m. morsitans*	Tanganyika Terr (Morogoro, Uluguru)	1915	Dr. A. G. Wilkins	M	1
	Tanganyika (Korogwed Handeni)	1952	16-IX-52 Brit. Mus. 1959-638 Dr. E. Burtt	M	1
	Tanganyika Terr: (Morogoro, Uluguru)	1921	Dr. A.G. Wilkins Pres. by Imp. Bur. Ent. Brit. Mus. 1921-152.	M	2
*G. m. centralis*	Tanganyika Terr.	1923	Brit. Mus. 1923-269	M	1
	Sedamara (Mbulu)	1950	26.9.50 London School of Hygiene & Tropical Medicine coll. BMNH	M	1
*G. p. gambiensis*	Sierra Leone (Scarcies, Kambia)	1946	Nash & Walton, 26/1/46	M	1
total					7
*G. pallidipes*	Ethiopia (Arba Minch)	2014		F	30
	Uganda (Lukoma – Bavuma)	2013		F	27
	Kenya (BioRI-KALRO)	2008		F	3
	Zambia (Mfuwe)	2007		F	3
	Zimbabwe (Ruckomechi)	2006		F	3
	Zimbabwe (Makuti)	2006		F	1
	Tanzania (Tanga)	2005		F	2
*G. m. morsitans*	Zambia (Mfuwe)	2007		F	1
	Zimbabwe (Ruckomechi)	2006		F	1
	Zimbabwe (Makuti)	2006		F	1
	Tanzania (Usinge)	2013		F	9
	Kenya (BioRI-KALRO)	2008		F	1
*G. m. centralis*	Angola (Guissakina)	2013		F	25
	Tanzania (Ugalla)	2013		F	60
*G. m. submorsitans* ^*b*^	Burkina Faso (Comoe)	2009		F	277
*G. p. gambiensis* ^*b*^	Senegal (Sebikotane)	2009		F	3
	Senegal (Sebikotane)	2013		F	9
	Senegal (Kayar)	2010		F	3
	Senegal (Kayar)	2013		F	17
	Senegal (Niokolo-Koba)	2012		F	3
	Senegal (Niokolo-Koba)	2013		F	30
	Senegal (Pout)	2009		F	11
	Senegal (Pout)	2013		F	30
	Burkina Faso (Comoe)	2008		F	1152
	Mali	2010		F	8
	Guinea	2010		F	1
*G. f. quanzensis*	Angola (Guissakina)	2013		F	3
	Uganda	2013		F	52
*G. brevipalpis*	Mozambique (Maputo GR)	2013		F	6
*G. swynnertoni* ^*b*^	Tanzania (Ikorongo GR)	2015		F	24
*G. medicorum*	Burkina Faso (Comoe)	2009		F	86
*G. tachinoides* ^*b*^	Burkina Faso (Comoe)	2009		F	792
	Ghana	2009		F	7
*G. austeni*	Mozambique (Maputo G)	2013		F	7
	Tanzania (Jozani)	1994		F	1
	Zanzibar (Unguja island)	1995		F	5
	South Africa (Zululand)			F	1
total					2695

*N* Number of specimens tested

*N/A* not available, *CIRDES* Centre International de Recherche-Développement sur l'Elevage en zone Subhumide, Bobo Dioulasso, Burkina Faso, *IPCL* Insect Pest Control Laboratory

^a^Type: L = Laboratory colony; M = Museum specimen; F = Field collection

^b^these collections included false assigned individuals (see also Table 4)

#### Museum specimens

*Glossina* specimens were obtained from Mr. Nigel P. Wyatt, Department of Entomology, Natural History Museum, London, UK (loan no. 2011-159) and comprised of representatives of the following *Glossina* taxa: *G. morsitans morsitans, G. morsitans centralis,* and *G. palpalis gambiensis*. These specimens were collected between 1915 and 1952 and were assigned to the respective taxa based on morphological characters (Table 1).

#### Natural populations

A total of 2634 individual tsetse flies, representing 30 taxon/geographical locations combinations from five countries in West Africa (Burkina Faso, Ghana Guinea, Mali, and Senegal), were included in this analysis. These samples were collected in different periods from 1994 to 2014 (Table 1) and were used as a ‘blind test’ to verify their species status using the ITS1 PCR amplicons, plus the *Wolbachia* infection, where necessary/applicable.

### DNA extraction, PCR, and sequencing

*Flies derived from laboratory colonies and natural populations*

DNA from teneral adult flies of each laboratory colony was isolated using the Qiagen DNeasy kit (Qiagen, Valencia, CA), following the manufacturer’s instructions. DNA samples were stored at 4 ^o^C until their use and at -20 ^o^C for long term. Samples collected from the field were sorted by species, labelled, kept in 95% ethanol (or propylene-1,2-diol), and shipped to the IPCL for downstream analysis. DNA extraction was performed as described for the laboratory colonies. For all PCR amplifications, 1.1X pre-aliquoted PCR master mix was used (ABgene, UK). In 22.5 μl of the mix, 1.5 μl of DNA template and 1μl of forward and reverse primer were added (10μM each). Nuclear (ITS1 and microsatellite), mitochondrial (*COI*, 16S *rRNA,* and 12S *rRNA*), and symbiotic markers (*Wolbachia* 16S *rRNA* gene) that were used in the present study are shown in Table 2. PCR conditions to amplify COI, 16S rRNA and ITS1 genes were as described previously [16]. Primers 12SCFR and 12SCRR were used to amplify a 377 bp fragment of the *12S rRNA* mitochondrial gene, as described in previous publications [52]. PCR conditions to detect the presence of cytoplasmic or nuclear *Wolbachia* 16S rRNA were as described previously using the *Wolbachia* specific primers wspecF and wspecR [52]. PCR conditions for the different sets of microsatellite markers were as described in the respective publications [16, 19, 21, 22, 53, 54]. PCR products were analysed on 1.5% agarose gels by electrophoresis and visualized using ethidium bromide. Amplicons of the mitochondrial genes were purified using QIAquick PCR kit (Qiagen Valencia, CA) and sequenced by MWG (MWG-Biotech AG, Germany). Forward and reverse sequences with good quality read were assembled and aligned using SeqMan Pro software (Lasergene 7.0, Dnastar Inc). The consensus sequences for each gene were aligned and trimmed using the ClustalW algorithm in MEGA version 6.0.

**Table 2:** A list of the molecular markers and primers used in this study

Molecular marker	Marker		Primer name	Primer sequence 5’-3’	Reference	Method of analysis
Nuclear markers	ITS1		*Glossina*ITS1_for	GTGATCCACCGCTTAGAGTGA	(Dyer *et al.*, 2008) [16]	Gel electrophoresis
			*Glossina*ITS1_rev	GCAAAAGTTGACCGAACTTGA		
	Microsatellite markers	A10	A10 F	GCAACGCCAAGTGAAATAAAG		
			A10 R	TACTGGGCTCGCGTACATAAT		
		Gmm14	Gmm14 F	CACACCCTGGATTACAAA	(Baker & Krafsur, 2001) [19]	
			Gmm14 R	TGAAATGCAACCCTTCTT		
Mitochondrial markers	COI		COI	TTGATTTTTTGGTCATCCAGAAGT	(Dyer *et al.*, 2008) [16]	Sequencing
			CULR	TGAAGCTTAAATTCATTGCACTAATC		
	16S rRNA		NI-J-12585	GGTCCCTTACGAATTTGAATATATCCT		
			LR-N-12866	ACATGATCTGAGTTCAAACCGG		
	12S rRNA		12SCFR	GAGAGTGACGGGCGATATGT	(Doudoumis *et al.*, 2012) [52]	
			12SCRR	AAACCAGGATTAGATACCCTATTAT		
Symbiotic markers	Wolbachia	16S rRNA	WspecF	YATACCTATTCGAAGGGATAG		Gel electrophoresis
			WspecR	AGCTTCGAGTGAAACCAATTC		

#### Museum specimens

Before DNA extraction, *Glossina* specimens were surface-sterilized by immersing in 80% ethanol and then rinsed with sterile PBS twice. DNA was extracted using Nucleospin Tissue Kit (Macheray-Nagel) following the manufacturer's instructions. DNA integrity was assayed by amplifying part of the mitochondrial 12S *rRNA* gene as described above. DNA samples were stored at 4 ^o^C until their use and at -20 ^o^C for long term storage. PCR amplifications were performed in reactions containing 10 ng DNA, 10 pmol of each primer, 0.5 units KAPA Taq (KAPA Biosystems), 1x KAPA buffer A (KAPA Biosystems), 0.25 mM deoxynucleotide triphosphate mixture (dNTPs) and water to a final volume of 20 μl. Amplification was performed in a PTC-200 Thermal Cycler (MJ Research), using the following cycling conditions: 95°C for 5 min, followed by 40 cycles of 30 s at 95 °C, 30 s at 54 °C, 1 min at 72 °C and a final extension of 10 min at 72 °C. PCR reactions were electrophoresed on a 1.5% agarose gel. Negative samples were reamplified by PCR using 2 μl of the first PCR reaction as template and the same set of primers and conditions for 35 cycles. Positive samples of the first or the second PCR reaction were further analyzed by double stranded sequencing with both forward and reverse primers. A dye terminator-labelled cycle sequencing reaction was conducted with the BigDye Terminator v3.1 Cycle Sequencing Kit (PE Applied Biosystems). Reaction products were analyzed using an ABI PRISM 310 Genetic Analyzer (PE Applied Biosystems). Gene sequences generated in this study were assembled and manually edited with SeqManII by DNAStar (Lasergene). For each sample, a majority-rule consensus sequence was created.

### Phylogenetic analysis

Phylogenetic analysis was performed using MEGA 6.0 software [55], using Maximum-Likelihood (ML) based on the General Time Reversible model with gamma distributed rates with 1000 bootstrap replications. *Musca domestica* (L.) sequences, which are closely related to *Glossina* genus, were used as outgroup for each of the analysed genes (gi|514058521, COI; AY573084.1, 12S rRNA).

## Results

### Evaluation of the discriminating power of different molecular tools

For the initial evaluation of the available molecular tools, ten laboratory colonies were used and eight to twelve individuals were genotyped per colony (Table 1).

#### Mitochondrial markers: COI and 16S rRNA

**Fig. 1:**
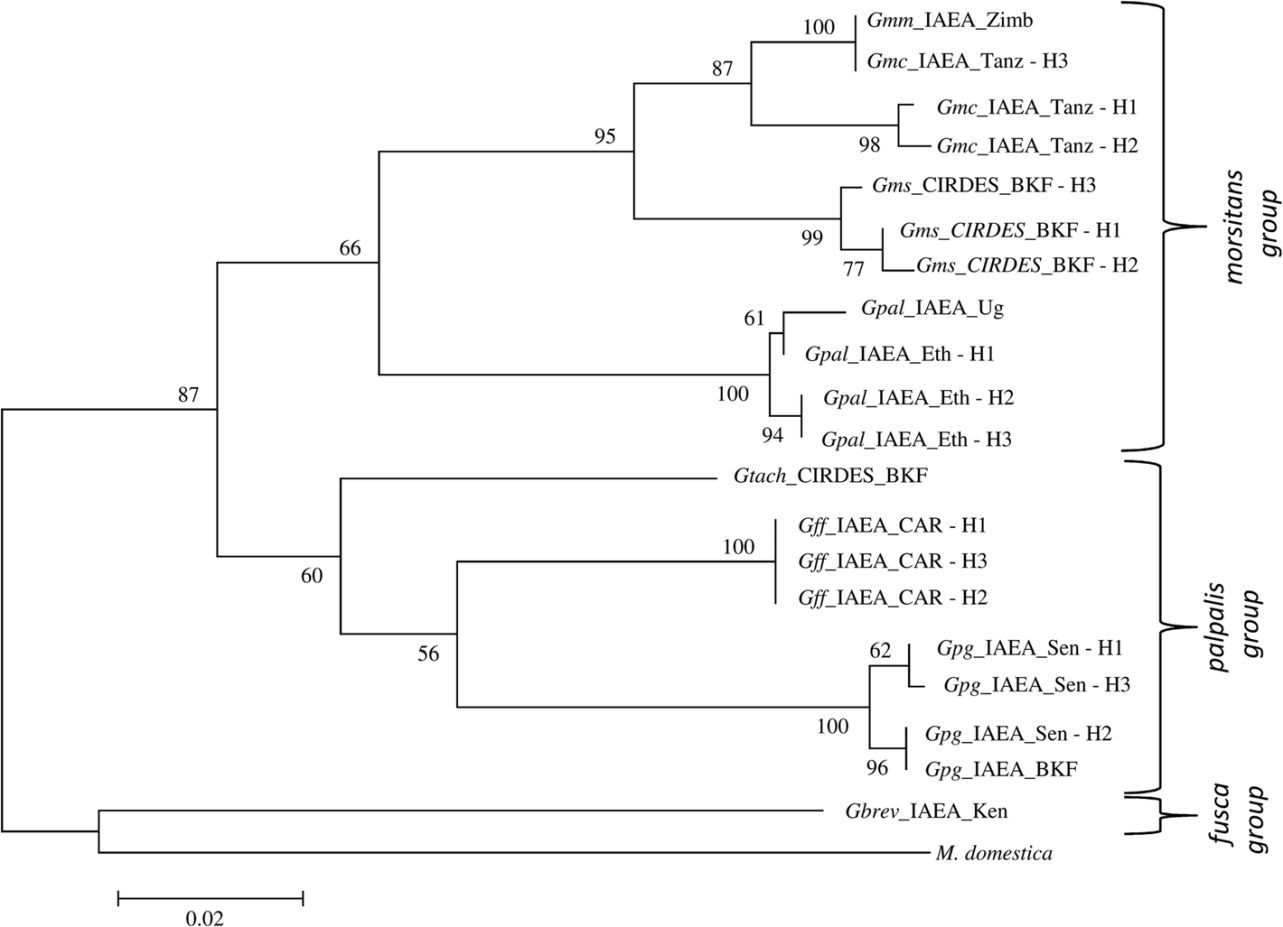
Molecular Phylogenetic analysis of laboratory populations by Maximum Likelihood method, using a COI gene fragment. The evolutionary history was inferred by using the Maximum Likelihood method based on the Tamura-Nei model. The tree with the highest log likelihood (-2065.3726) is shown. The percentage of trees in which the associated taxa clustered together is shown next to the branches. Initial tree(s) for the heuristic search were obtained automatically by applying Neighbor-Join and BioNJ algorithms to a matrix of pairwise distances estimated using the Maximum Composite Likelihood (MCL) approach, and then selecting the topology with superior log likelihood value. The tree is drawn to scale, with branch lengths measured in the number of substitutions per site. The analysis involved 20 nucleotide sequences. Codon positions included were 1st+2nd+3rd+Noncoding. All positions containing gaps and missing data were eliminated. There was a total of 600 positions in the final dataset. *Musca domestica* was used as outgroup. The numbers at each node represent bootstrap proportions based on 1000 replications. All abbreviations used in the Figures are shown in Additional file 5.

Sequence datasets generated for each of the mitochondrial genes (616 bp for *COI* and 207 bp 16S *rRNA*) were aligned for all ten *Glossina* laboratory colonies. The phylogenetic reconstruction for each of the mitochondrial markers clearly clustered the three taxonomic groups of *Glossina* (*palpalis, morsitans* and *fusca* groups). *COI* was more informative than 16s *rRNA* and was selected as a representative gene of the mitochondrial DNA (Fig. 1). However, clustering in sub species and closely related species level was not always accurate, as in the case of *G. m. morsitans* and *G. m. centralis*. Within some taxa, distinct haplotypes were observed using either the *COI* gene (Fig. 1) or the 16S *rRNA* gene (data not shown). For instance, *G. m. centralis, G. pallidipes* from Ethiopia, *G. f. fuscipes,* and *G. p. gambiensis* from Senegal were found to have three haplotypes each (H1, H2, H3) for the *COI* dataset.

#### Nuclear markers: ITS1 and microsatellite markers

**Fig. 2:**
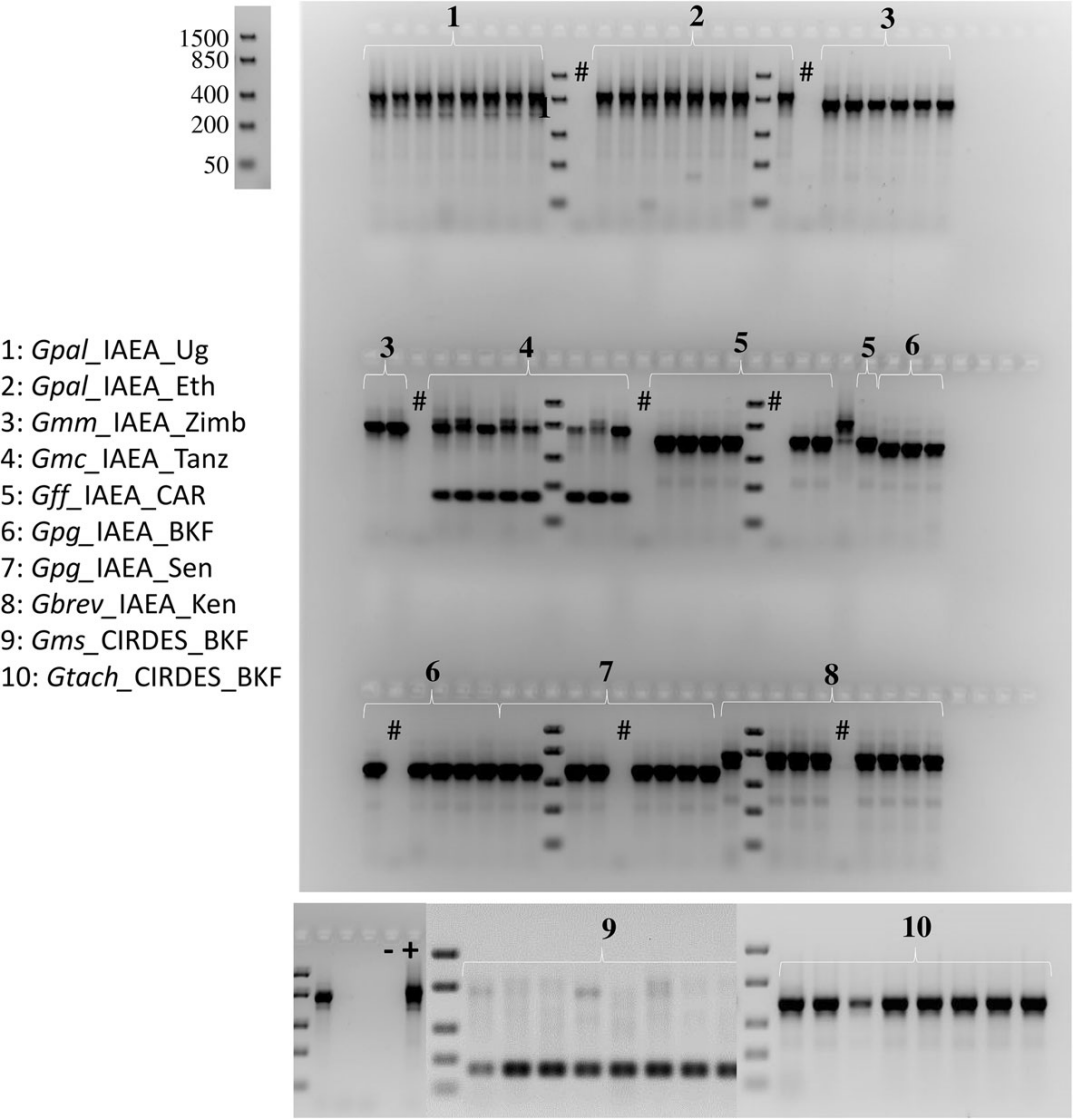
Agarose gel electrophoresis (2.5% agarose) showing the ITS1 gene amplicons for the different tsetse laboratory populations. Eight to twelve flies per laboratory population were analyzed. All abbreviations used in the Figures are shown in Additional file 5. The DNA ladder used to determine the size of the analyzed PCR products is also shown. #: Negative control during DNA extraction; -: negative PCR control; +: positive PCR control (*G. pallidipes* DNA).

Variation in the length of the ITS1 amplicon was observed across the different *Glossina* laboratory colonies, consistent with the species identification (Table 3, Fig. 2, Additional files 1, 2 and 3). Based on size and/or number of amplicons, as revealed by agarose gel electrophoresis, most of the taxa were successfully separated. Among eight screened taxa, only *G. m. centralis/G. m. submorsitans* and *G. m. morsitans/G. brevipalpis* could not be separated from each other. However, sequencing analysis showed that there was a three bp difference between the amplicons of *G. brevipalpis* (778 bp) and *G. m. morsitans* (775 bp). This difference can be used to differentiate among them, using higher resolution fragment analysis approaches, such as polyacrylamide gel, low melting agarose or capillary electrophoresis. To further evaluate the discriminative power of ITS1, field collection representing *G. swynnertoni* (Tanzania) was added in this analysis. This sample shared the ITS1 pattern of the *G. m. morsitans/G. brevipalpis* group (~775 bp) (Fig. 2).

**Table 3:** Analysis of ITS1 sequence length, microsatellite markers and *Wolbachia* status in *Glossina* laboratory populations

*Glossina* species	Country of origin (Location)	No	ITS1 expected size	Wolbachia		Microsatellites		Correctly identified samples
				cytoplasmic	chromosomal	A10	Gmm14	
*G. pallidipes*	IPCL Uganda	8	920	0.0 % (0/8)	0.0 % (0/8)	-	+	8/8
	IPCL Ethiopia	8		12.5 % (1/8)	0.0 % (0/8)	-	+	8/8
*G. m. morsitans*	IPCL	8	775	75 % (6/8)	100 % (8/8)	-	+	8/8
*G. m. centralis*	IPCL	8	~800 + ~150	100 % (8/8)	0.0 % (0/8)	-	+	8/8
*G. m. submorsitans*	CIRDES	12	~800 + ~150	0.0 % (0/12)	0.0 % (0/8)	-	+	12/12
*G. p. gambiensis*	IPCL POUT	8	543	0.0 % (0/8)	0.0 % (0/8)	+	+	8/8
	IPCL CIRDES	8		0.0 % (0/8)	0.0 % (0/8)	+	+	8/8
*G. f. fuscipes*	IPCL Central Africa Republic	8	618	12.5 % (1/8)	0.0 % (0/8)	Partial	+	8/8
*G. brevipalpis*	IPCL	8	778	75 % (6/8)	0.0 % (0/8)	-	-	8/8
*G. tachinoides*	CIRDES	12	597	0.0 % (0/12)	0.0 % (0/8)	-	+	12/12

-: no amplicon detected

+: the expected amplicon was detected in all individuals screened

Partial: the expected amplicon was detected, but not in all individuals screened

**Fig. 3:**
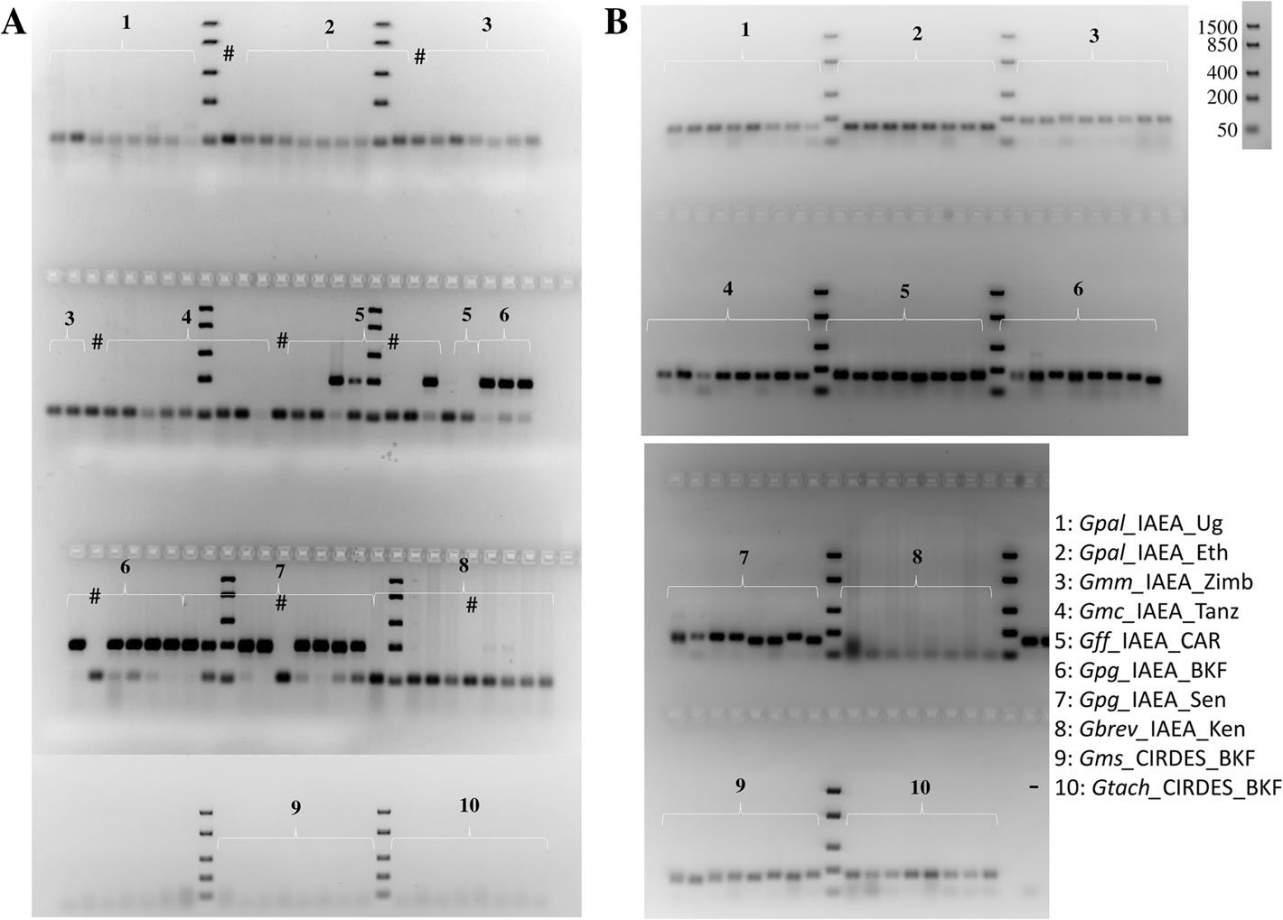
Agarose gel electrophoresis (2% agarose) presenting microsatellite markers A10 (**a**) and Gmm14 (**b**) amplicons for the different laboratory populations. Eight to twelve flies per laboratory population were analyzed. All abbreviations used in the Figures are shown in Additional file 5. The DNA ladder used to determine the size of the analyzed PCR products is also shown. #: Negative control during DNA extraction; -: negative PCR control

A set of 36 previously published microsatellite markers was tested against 1-3 individuals of the ten laboratory populations (Additional file 1). The analysis was carried out only with agarose gel electrophoresis and showed that there are microsatellite markers producing species-specific amplicons in the expected size range. As an example, microsatellite marker A10, which had been designed for *G. f. fuscipes* and was reported to be specific for *G. p. gambiensis,* produced the expected amplicon in all *G. p. gambiensis* specimens plus some of the *G. f. fuscipes* samples but gave no amplicons in all other taxa (Fig. 3a). Also, microsatellite marker Gmm14 amplified in all taxa analyzed except *G. brevipalpis* (Fig. 3b).

#### Wolbachia 16S rRNA

**Fig. 4:**
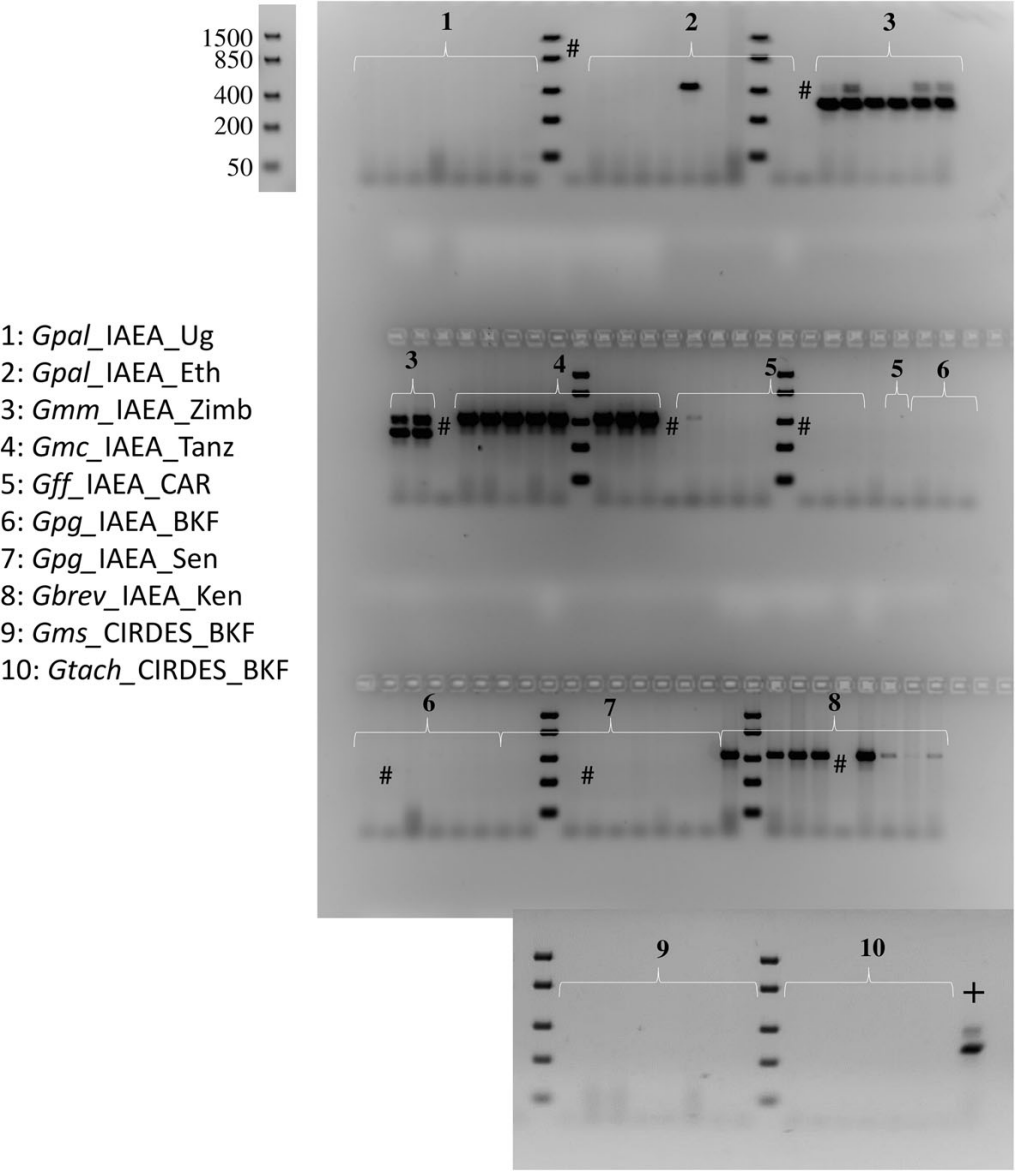
Agarose gel electrophoresis (2% agarose) showing the *Wolbachia* amplicons for the different laboratory populations. The presence of the 438 bp amplicon is indicative of an active (cytoplasmic) *Wolbachia* infection, while the 296 bp amplicon is indicative of the presence of the partial sequence of the *Wolbachia* 16S *rRNA* gene that is integrated into the tsetse genome. Eight to twelve flies per laboratory population were analyzed. All abbreviations used in the Figures are shown in Additional file 5. The DNA ladder used to determine the size of the analyzed PCR products is also shown. #: Negative control during DNA extraction; +: positive PCR control (*G. m. morsitans* DNA).

The prevalence of *Wolbachia* infections differed significantly between the different laboratory colonies (Additional file 4). A fixed cytoplasmic *Wolbachia* infection (with strong PCR amplicons) was detected only in *G. m. centralis.* High infection prevalence (with strong PCR amplicons) was observed in *G. brevipalpis* and *G. m. morsitans*. Sporadic infections (with weak PCR amplicons) were observed in *G. pallidipes* and *G. f. fuscipes.* However, *G. m. morsitans* presented the fixed chromosomal insertion (296 bp amplicon) previously reported [52] that was present in none of the other laboratory colonies. The remaining taxa/colony (*G. m. sub-morsitans, G. p. gambiensis,* and *G. tachinoides*) did not give any amplicon indicative of either active cytoplasmic infection or chromosomal insertion of *Wolbachia* (Table 3, Fig. 4).

### Correlation with museum specimens

**Fig. 5:**
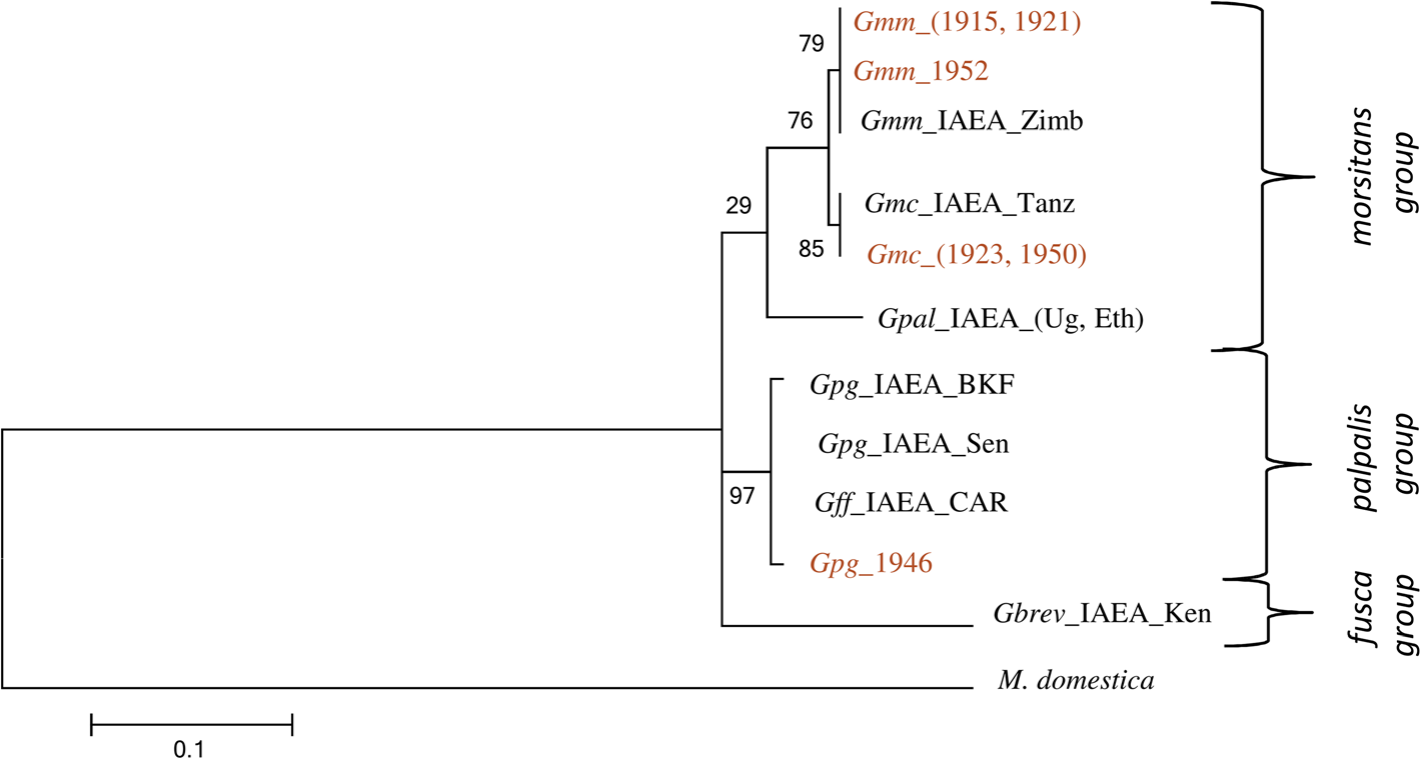
Molecular Phylogenetic analysis of laboratory populations and museum specimens by Maximum Likelihood analyses, using the 12S *rRNA* gene sequence. The evolutionary history was inferred by using the Maximum Likelihood method based on the Tamura-Nei model. The tree with the highest log likelihood (-629.9965) is shown. The percentage of trees in which the associated taxa clustered together is shown next to the branches. Initial tree(s) for the heuristic search were obtained automatically by applying Neighbor-Join and BioNJ algorithms to a matrix of pairwise distances estimated using the Maximum Composite Likelihood (MCL) approach, and then selecting the topology with superior log likelihood value. The tree is drawn to scale, with branch lengths measured in the number of substitutions per site. The analysis involved 12 nucleotide sequences. Codon positions included were 1st+2nd+3rd+Noncoding. All positions containing gaps and missing data were eliminated. There was a total of 180 positions in the final dataset. The numbers at each node represent bootstrap proportions based on 1000 replications. Laboratory populations are in black and Museum specimens are in brown. *Musca domestica* was used as outgroup. All abbreviations used in the Figures are shown in Additional file 5.

Due to low DNA quality, only few amplicons were obtained from museum specimens and only for the 12S *rRNA* gene. Therefore, representative samples from all laboratory colonies were also sequenced for the 12S *rRNA* gene. Despite the limited resolution provided, the laboratory colonies correlated well with the museum specimens (Fig. 5).

### Evaluation of COI as a ‘stand-alone’ marker for species identification

**Fig. 6:**
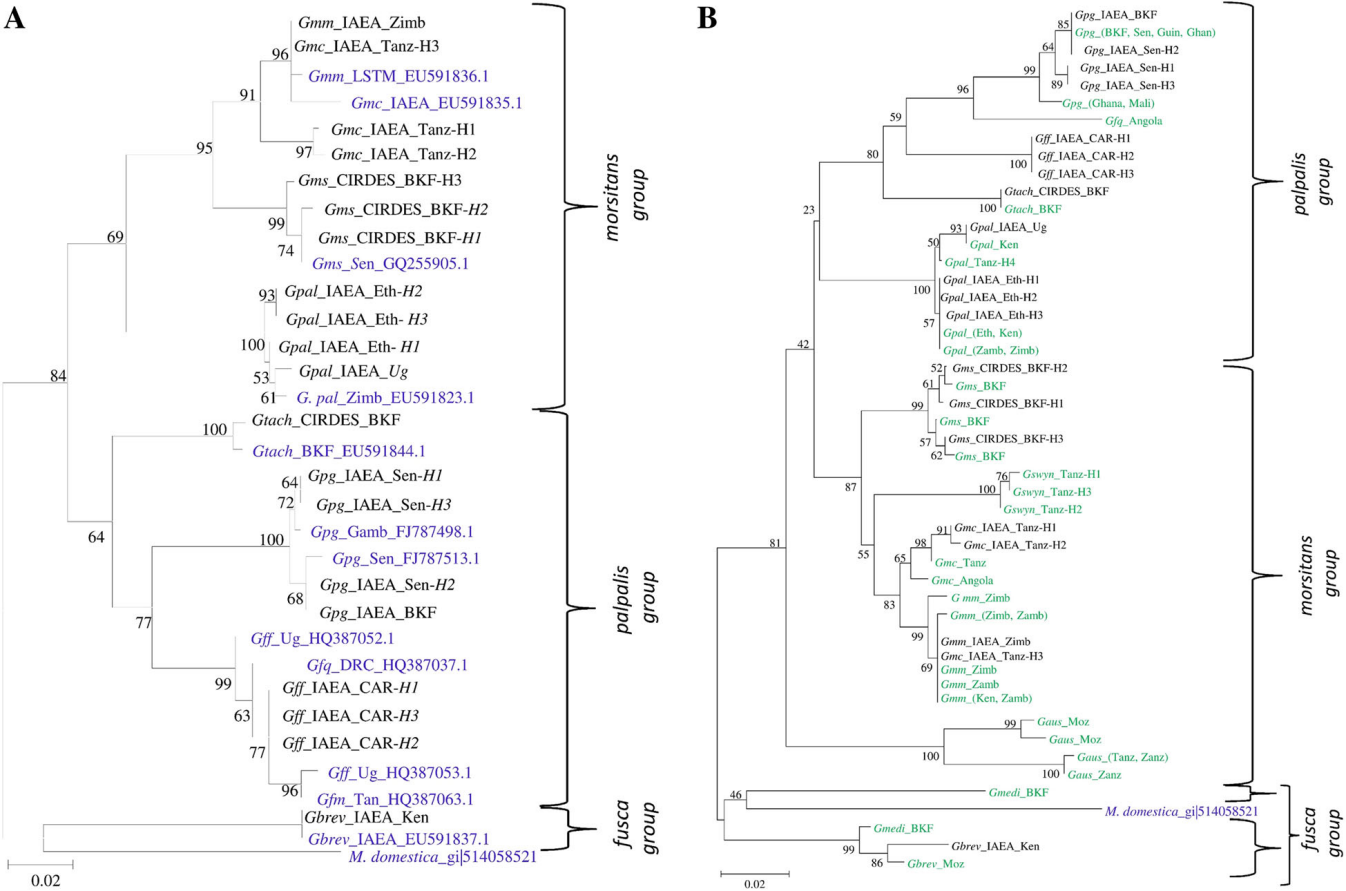
Molecular Phylogenetic analysis of laboratory populations, published sequences, and selected samples from collections deriving from wild (**b**), by Maximum Likelihood method. Using a *COI* gene fragment. **a** The evolutionary history was inferred by using the Maximum Likelihood method based on the Tamura-Nei model. The tree with the highest log likelihood (-2609.6833) is shown. The percentage of trees in which the associated taxa clustered together is shown next to the branches. Initial tree(s) for the heuristic search were obtained automatically by applying Neighbor-Join and BioNJ algorithms to a matrix of pairwise distances estimated using the Maximum Composite Likelihood (MCL) approach, and then selecting the topology with superior log likelihood value. The tree is drawn to scale, with branch lengths measured in the number of substitutions per site. The analysis involved 33 nucleotide sequences. Codon positions included were 1st+2nd+3rd+Noncoding. All positions containing gaps and missing data were eliminated. There was a total of 600 positions in the final dataset. Samples derived from laboratory populations of the present study are in black and different tsetse sequences available in the NCBI database are in blue. *Musca domestica* was used as outgroup. All abbreviations used in the Figures are shown in Additional file 5. **b** The evolutionary history was inferred by using the Maximum Likelihood method based on the Tamura-Nei model. The tree with the highest log likelihood (-2044.8169) is shown. The percentage of trees in which the associated taxa clustered together is shown next to the branches. Initial tree(s) for the heuristic search were obtained automatically by applying Neighbor-Join and BioNJ algorithms to a matrix of pairwise distances estimated using the Maximum Composite Likelihood (MCL) approach, and then selecting the topology with superior log likelihood value. The tree is drawn to scale, with branch lengths measured in the number of substitutions per site. The analysis involved 49 nucleotide sequences. Codon positions included were 1st+2nd+3rd+Noncoding. All positions containing gaps and missing data were eliminated. There was a total of 362 positions in the final dataset. *Musca domestica* was used as outgroup. All abbreviations used in the Figures are shown in Additional file 5. Samples derived from laboratory populations of the present study are in black and samples collected from the field are in green.

*COI* gene sequence was used to a) correlate our reference laboratory colonies with published sequences of different taxa, and b) identify selected samples from the field that were available in IPCL DNA base. In general, laboratory colonies were well correlated both to previously published sequences (Fig. 6a) and samples field collections available in our DNA base (Fig. 6b). On the other hand, *COI* cannot clearly resolve closely related species (subspecies or complex species), as was the case of the *G. morsitans* subspecies and *G. f. quanzensis* from Angola (which is more closely related to the *G. p. gambiensis* samples, rather than the rest of the *G. fuscipes* samples (Fig. 6b).

### Development of a multi-marker species identification approach

**Fig. 7:**
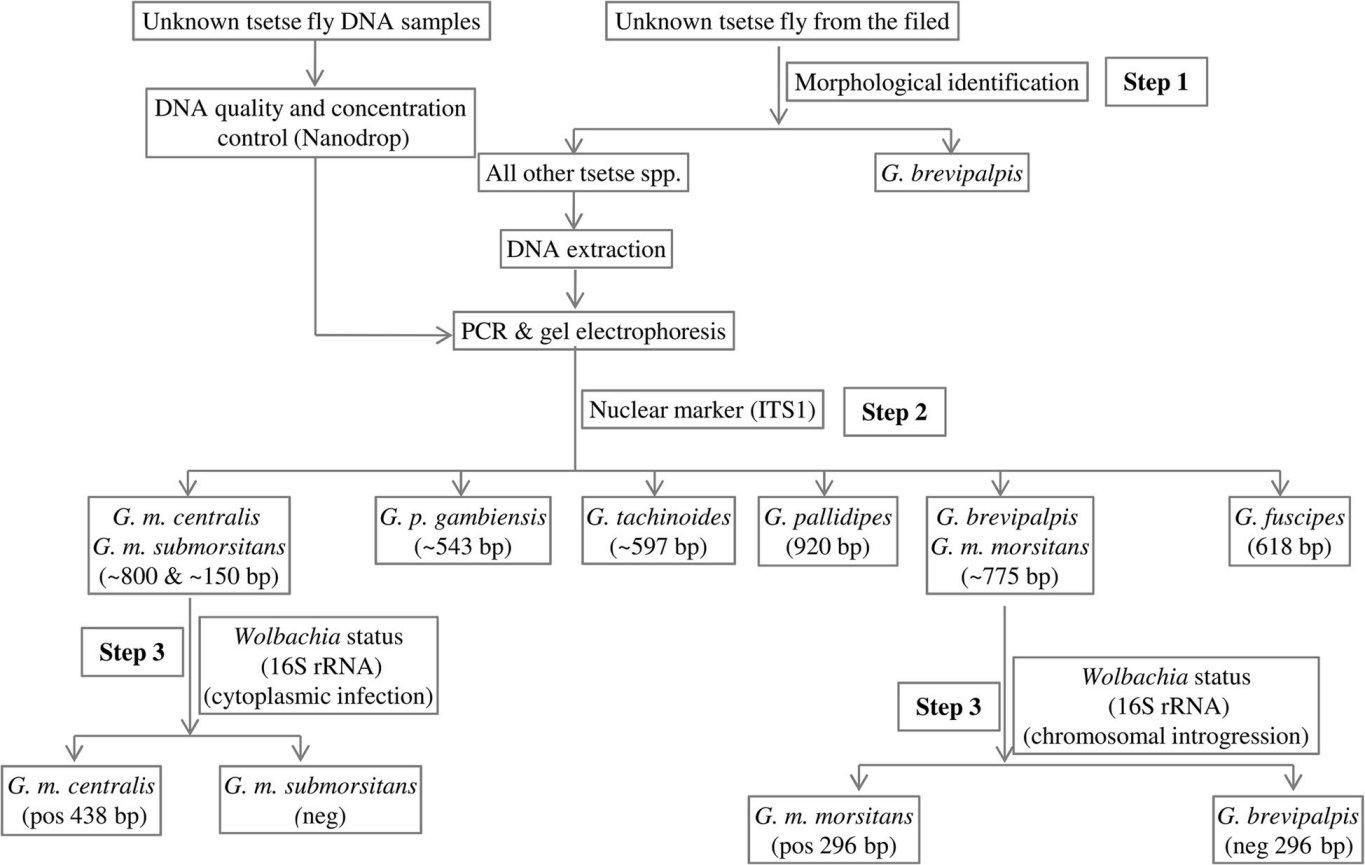
A multi-marker based approach to distinguish tsetse species, based on agarose gel electrophoresis. This approach relies on the amplicons (size and number) of ITS1 and the presence/absence of the *Wolbachia* specific *16S rRNA* amplicons (both cytoplasmic and chromosomal)

Based on the initial data derived from the laboratory colonies, we focused on the discriminative power of the combined use of ITS1, microsatellite markers Gmm14/A10, and the *Wolbachia* status (both cytoplasmic and chromosomal), utilizing only agarose gel electrophoresis. Previous findings as well as the findings of this study, suggested that the length of the ITS1 amplicon should be sufficient to identify most of the taxa analyzed, except two cases: the *G. m. centralis/G. m. submorsitans* group and the *G. m. morsitans/ G. brevipalpis* (Fig. 2)*.* To differentiate *G. m. centralis* from *G. m. submorsitans*, we used the *Wolbachia* infection status (cytoplasmic) (Fig. 4). To differentiate *G. m. morsitans* from *G. brevipalpis*, we used the *G. m. morsitans* – specific chromosomal introgression of the *Wolbachia* 16S *rRNA* gene (Fig. 4). These results are summarized in Table 3 and the approach used to differentiate among the available taxa is summarized in Fig. 7. Following this approach, without using any morphological data, all ten laboratory colonies (representing 8 taxa) were accurately resolved.

### The ‘blind test’ using ITS1, selected microsatellite markers, and Wolbachia

To further test the resolution power of this combined approach, a ‘blind test’ of randomly selected DNAs available at the DNA base of the IPCL was performed. The first step was the application of the ITS1 assay. A total of 2695 individuals were genotyped and 2662 (98.78 %) were assigned to the expected taxon (Table 4), based on the information available upon collection. For 33 individuals, there was a discrepancy between data obtained upon collection and ITS1 profile. More specifically, for 0.57 % of the *G. p. gambiensis* samples (7 out of 1267), 7.94 % of the *G. m. submorsitans* samples (22 out of 277), 0.13 % of the *G. tachinoides* samples (1 out of 799), and 12.5 % of the *G. swynnertoni* samples (3 out of the 24), data from collection sites were not in agreement with the molecular identification (Table 4). These samples were revisited and the *Wolbachia* infection status, the amplicon profile of microsatellite markers A10 and Gmm14, and the sequencing data of *COI* gene were also used. The combined use of the four classes of markers, along with data of the geographical distribution of *Glossina* species verified the taxon of these samples, showing that they were cases of either misidentification in the field or subsequent mislabeling (Table 4). Therefore, all samples were correctly identified with the combined use of these markers. In this analysis, four field collected samples representing four additional taxa were included (*G. austeni, G. f. quanzensis, G. medicorum,* and *G. swynnertoni*)*.* For these taxa, there were no laboratory colonies available to use as reference. The estimated size of ITS1 amplicons were in accordance with that expected from previous studies. The pattern of ITS1 is sufficient to differentiate both *G. austeni* (amplicon of 633 bp) from all other taxa of this study, although this amplicon size is very similar to the *G. fuscipes* amplicon size (633 bp). *G. f. quanzensis* could not be differentiated from *G. f. fuscipes*, based on the single agarose gel electrophoresis of the ITS1 amplicon. *G. medicorum* gave two amplicons, with the one having a size between 600 and 700 bp, and the other being close to the one expected from previous studies (~880 bp). However, in our samples, the amplicon of lower molecular weight (600 -700 bp) was more robust and consistent than the expected one. *G. swynertoni* provided a unique combined profile: (a) the COI sequencing data place these samples close to *G. m. centralis* and *G. m morsitans* (Fig. 6b, Additional file 5), (b) the ITS1 profile (amplicon size) is similar or identical to *G. m. morsitans* and *G. brevipalpis* and (c) the *Wolbachia* infection status (complete absence of both cytoplasmic and chromosomal amplicons). Due to the lack of reference laboratory colonies, the *G. swynertoni* samples were not included in the approach described in Fig. 7.

**Table 4:**
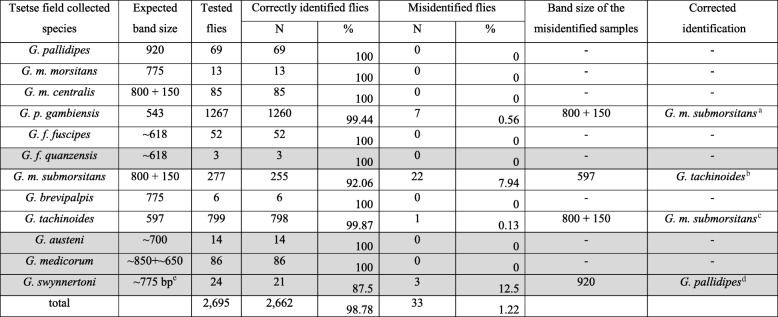
Validation of Tsetse species from field collected samples using Glossina ITS1

In grey scale: field collections lacking reference laboratory populations

^a^Based on the ITS1 profile, non-amplification of microsatellite A10, complete absence of the cytoplasmic infection of *Wolbachia,* and the geographical distribution of tsetse species, these 7 samples were identified as *G. m. submorsitans*

^b^Based on the ITS1 profile, amplification pattern of both A10 and Gmm14 microsatellite markers, absence of cytoplasmic and chromosomal *Wolbachia*, and the geographic distribution of tsetse species, these individuals were identified as *G. tachinoides*

^c^Based on the ITS1 profile, amplification of both A10 and Gmm14 microsatellite, absence of cytoplasmic and chromosomal *Wolbachia*, and the geographic distribution of tsetse species, these individuals were identified as *G. m. submorsitans*

^d^Based on the ITS1 profile, COI profile, amplification pattern of both A10 and Gmm14 microsatellite markers, absence of cytoplasmic and chromosomal *Wolbachia*, and the geographic distribution of tsetse species, these individuals were identified as *G. pallidipes*

^e^For *G. swynnertoni*, there was no ITS1 amplicon expected from previous studies. The one generated in the present study is stated as ‘expected’

Of special interest is the combined use of ITS1 and *Wolbachia* to differentiate among the subspecies of *G. morsitans.* As described, *G. m. morsitans* has a distinct ITS1 profile and the presence of the chromosomal introgression of *Wolbachia. G. m. centralis* and *G. m submorsitans*, which share the same characteristic ITS1 pattern, can be differentiated by the presence of an active *Wolbachia* infection. To support this, 85 field collected individuals belonging to *G. m. centralis* (Angola and Tanzania), that had the same ITS1 profile, were also 100 % infected with *Wolbachia* (Table 5)*.* Regarding *Wolbachia* status of the other field collected samples, *G. austeni* was 100 % infected, *G. brevipalpis* did not show a fixed infection pattern (in a small sample size though with strong PCR amplicons in some of the individuals), and three other taxa also presented non-fixed infection patterns and with weak PCR amplicons (*G. f. fuscipes, G. f. quanzensis,* and *G. p. gambiensis*). *G. pallidipes* did not show any evidence of *Wolbachia* infection (Table 5).

**Table 5:** *Wolbachia* status of selected *Glossina* field collections

Field collected tsetse species	Wolbachia status				
	Cytoplasmic			Chromosomal	
	*N*	%	Estimation	*N*	%
*G. pallidipes*	0/57	0	no PCR amplicon, no infection	0/57	0
*G. m. centralis*	85/85	100	strong PCR amplicons, fixed infection	0/85	0
*G. p. gambiensis*	15/78	19.2	weak PCR amplicons, sporadic	0/78	0
*G. f. fuscipes*	2/52	3.8	weak PCR amplicons, sporadic	0/52	0
*G. f. quanzensis*	1/3	33.3	weak PCR amplicons, sporadic	0/3	0
*G. brevipalpis*	3/6	50	strong PCR amplicons, not fixed infection	0/6	0
*G. austeni*	7/7	100	strong PCR amplicons, fixed infection	0/7	0

## Discussion

The present study clearly suggests that the combined use of ITS1, selected microsatellite markers, and *Wolbachia* status (cytoplasmic infection and chromosomal introgression) provides a reliable and cost-effective approach that can be applied for the identification of many *Glossina* taxa, without need of sequencing.

Sequencing of some of the mitochondrial genes supports the phylogeny of three *Glossina* groups. Different haplotypes within some species were revealed for the COI gene sequence. Although the sequencing of the mitochondrial markers showed differences among the *Glossina* species and even within populations of different geographical areas, these sequences alone could not distinguish among some taxa. For instance, the *G. m. centralis H3* COI and 16S *rRNA* gene sequences were similar to the *G. m. morsitans* sequences. Additionally, mitochondrial markers can be considered as ‘compromised’ in cases of closely related species. In such cases, mitochondrial haplotypes may have a completely different phylogenetic history than nuclear DNA. Moreover, the distinct patterns of *Wolbachia* infections in the different *Glossina* taxa make the use of mitochondrial markers even more questionable. For these reasons, sequencing of mitochondrial markers was not included as a tool in the approach followed in the present study.

The ITS1 sequence length variation proved quite a reliable marker in species level. The ITS1 amplicons generated from this study are in accordance with previously published ITS1 sequenced species [15–17] (Additional file 2). Some ITS1 amplicons, representing sequence variants from different taxa (from reference laboratory colonies only), were sequenced to confirm the actual amplicon size (data not shown). In all cases, sequences matched the published ITS1 sequences [16].

Taking together results from laboratory and field samples, the ITS1 amplicon produced eight size variants that could easily be recognized in 2.5 % agarose gel electrophoresis. These profiles successfully identified five species (*G. pallidipes, G. p. gambiensis, G. tachinoides, G. austeni,* and *G. medicorum*)*.* The three remaining ITS1 profiles clustered seven taxa in three different groups. The *G. m. morsitans / G. swynnertoni / G. brevipalpis* group, the *G. m. centralis / G. m. submorsitans group,* and the *G. f. fuscipes / G. f. quanzensis* group. To provide further analysis, several microsatellite markers were screened to identify some taxon-specific markers that could be used as diagnostic markers among specific taxa and we coupled this with ‘symbiotic markers’ that is the *Wolbachia* status. Cross-species amplification of microsatellite markers is an indication of the phylogenetic relation among different taxa and more closely related taxa are expected to share a higher number of cross amplified markers and this also can be regarded as an indicator of their genetic proximity. This property has been already exploited in *Glossina* species to avoid the *de novo* development of markers (Additional file 3). As previously reported [16], microsatellite A10 can be used to distinguish *G. p. gambiensis* from *G. tachinoides* which showed similar (but not identical) ITS1 length. Moreover, microsatellite Gmm14 can successfully differentiate *G. brevipalpis* from all other taxa in this study, which was crucial since it shared an identical (or similar) ITS 1 profile with *G. m. morsitans* and *G. swynnertoni*. The two remaining ‘black boxes’ are the *G. m. morsitans / G. swynnertoni* and the *G. m. centralis / G. m. submorsitans* groups. However, based on our (and previous) data, they can be separated based on the *Wolbachia* profile. *G. m. morsitans* is up to now the only taxon that has a *Wolbachia* chromosomal insertion that gives a characteristic 16S *rRNA* amplicon of 296 bp and *G. swynnertoni* samples tested did not produce this amplicon. Regarding the last group, *G. m. centralis* has a fixed *Wolbachia* infection (cytoplasmic), while *G. m. submorsitans* seems to lack *Wolbachia.* Regarding the *G. fuscipes* subspecies, we did not have well characterized material besides *G. f. fuscipes.* Few field collected individuals were available for *G. f. quanzensis* that shared the same ITS1 profile with *G. f. fuscipes.* Dyer and her colleagues have developed ITS1 diagnostic primer pairs and diagnostic assays that can differentiate among the three subspecies of *G. fuscipes (fuscipes, quanzensis,* and *martinii*) [17]. Since we did not have reference laboratory material for the two of the three subspecies, we could not investigate the identification of these taxa further.

Sequencing of *COI* gene and presence/absence of microsatellite amplicons of the selected microsatellite markers has not been included in our final combined approach (Fig. 7). *COI* gene sequencing has been excluded trying to keep the protocol cheap, quick, and easy to apply, taking into account also the reduced credibility of mitochondrial markers for the discrimination of taxa when: (a) there is gene flow among them and (b) there is documented presence of reproductive symbionts, such as *Wolbachia*. Microsatellite markers have been also excluded for different reasons. Although they gave clear results for selected taxa, we wanted to avoid including markers, which are based on the presence/absence of an amplicon using non-universal primers, since negative results are always difficult to evaluate and classify. Therefore, only the *Wolbachia* status relies on the presence/absence of an amplicon using universal *Wolbachia* 16S *rRNA* gene primers. However, including the microsatellite markers in the analysis can provide redundancy and increased robustness in the interpretation of the data. Moreover, they may be useful for the identification of taxa that have not been included in the present study.

Among the ten laboratory colonies screened here, only *G. m. centralis* harbored a fixed *Wolbachia* infection and only *G. m. morsitans* showed a fixed chromosomal insertion. All other laboratory colonies were shown to be either *Wolbachia-*free (*G. pallidipes, G. p. gambiensis, G. m. submorsitans,* and *G. tachinoides*) or had varying levels of *Wolbachia* infection (*G. m. morsitans*, *G. f. fuscipes,* and *G. brevipalpis*). These data are in agreement with previous studies about the *Wolbachia* infection status of laboratory colonies and natural populations of *Glossina* species [32, 41, 52, 56, 57]. The presence of *Wolbachia* in some of the *G. pallidipes* flies from Ethiopia and its absence from all Uganda *G. pallidipes* flies suggests that geographical origin of a species might impact the *Wolbachia* infection status of the species. The presence or absence of *Wolbachia* infection in the same species from different geographical areas has been previously reported [32, 41, 52, 56]; however, many of these cases are both low prevalence and low titer infections (Additional file 4). The biological, ecological and evolutionary significance of such infections remains to be resolved.

The horizontal gene transfer of *Wolbachia* was found fixed in *G. m. morsitans* laboratory colony, using the 16S *rRNA* gene-based PCR assay, in agreement with already published results [40, 41]. None of the other laboratory colonies and field collections of any other taxon showed evidence of the specific chromosomal insertion. We did not have material to expand our sampling of *G. m. morsitans* but all the material belonging to *G. m. centralis* and *G. m. submorsitans*, both laboratory and field collected, were negative.

## Conclusions

The integration of nuclear and symbiotic markers in this study could clearly discriminate among some different economically important *Glossina* taxa (Fig. 7). The correct identification at least at the species level is crucial for the application of SIT and requires large numbers of individuals, especially in cases of morphologically indistinguishable subspecies, complexes of species and sympatric species. We avoided using sequencing and/or specialized PCR assays (diagnostic primer pairs) to keep the identification test easy to apply, easy to analyze and of low cost. Although there are now modern tools available that can support molecular taxonomy (genome wide sequencing for example), they cannot yet be used cost effectively on numerous individuals. Therefore, our approach can be considered as adequate to support species identification, especially in African countries where quick decision making and planning may be needed, depending on the data derived from trap collections.

## Additional files

Additional file 1: The set of microsatellites markers tested for the identification of *Glossina* species. These markers were evaluated against different laboratory populations, considering the amplification of the expected PCR product. (DOCX 19 kb)

Additional file 2 ITS1 size variants as published in previous studies. (DOCX 17 kb)

Additional file 3: Microsatellite markers’ cross species amplification in different *Glossina* taxa as referred in previous publications. (DOCX 24 kb)

Additional file 4: *Wolbachia* status in different *Glossina* taxa as referred in previous publications. (DOCX 14 kb)

Additional file 5: Abbreviations used in the Figures (taxon name and country of origin). (DOCX 15 kb)

## Abbreviations

AAT: Animal African trypanosomosis
CI: Cytoplasmic Incompatibility
COI: Cytochrome oxidase 1
COII: Cytochrome oxidase 2
CYTB: Cytochrome b
HAT: Human African trypanosomosis
IPCL: Insect Pest Control Laboratory
ITS1: Internal Transcribed Spacer 1
ND2: NADH dehydrogenase 2
PBS: Phosphate Buffer Saline
SAT: Sequential Aerosol Technique
SIT: Sterile Insect Technique
