# Supplementary material for: Nuclear and Wolbachia-based multimarker approach for the rapid and accurate identification of tsetse species
Source: BMC Microbiol. 2018 Nov 23;18(Suppl 1):147. doi: 10.1186/s12866-018-1295-4 (PMC6251096; doi:10.1186/s12866-018-1295-4)
Supplement: Supplementary file 1 — The set of microsatellites markers tested for the identification of Glossina species. These markers were evaluated against different laboratory populations, considering the amplification of the expected PCR product. (DOCX 19 kb) [file 12866_2018_1295_MOESM1_ESM.docx]

**Supplementary Table S1:** **The set of microsatellites markers tested for the identification of *Glossina* species**. **These markers were evaluated against different laboratory populations, considering the amplification of the expected PCR product**

| **Microsatellite marker** | **Species** | | | | | | | |
| --- | --- | --- | --- | --- | --- | --- | --- | --- |
|  | *G. pallidipes* | *G. m. morsitans* | *G. m. centralis* | *G. m. submorsitans* | *G. tachnoides* | *G. brevipalpis* | *G. f. fuscipes* | *G. p. gambiensis* |
| GffA3 |  |  |  | **X** |  | **X** | **X** | **X** |
| GffA9 |  |  |  |  | **X** |  | **X** | **X** |
| GffB101 |  |  |  |  |  |  | **X** | **X** |
| GffA10 (or ‘A10’) |  | **X** |  |  |  | **X** |  | **X** |
| 69.22Gpg |  | **X** | **X** |  |  | **X** | **X** | **X** |
| GffB8 |  |  |  |  |  |  | **X** | **X** |
| GffA19a |  | **X** | **X** | **X** | **X** | **X** | **X** | **X** |
| GffA23b |  |  |  |  |  |  |  |  |
| GpB6b | **X** |  |  |  |  |  |  |  |
| GffA6 |  | **X** | **X** | **X** | **X** | **X** | **X** | **X** |
| Gpc107 | **X** | **X** | **X** | **X** | **X** | **X** | **X** | **X** |
| 55.3Gpg |  |  |  |  | **X** | **X** | **X** | **X** |
| 19.62Gpg |  |  |  |  | **X** | **X** | **X** | **X** |
| Gmm8 | **X** | **X** | **X** | **X** | **X** | **X** | **X** | **X** |
| Gmm14 | **X** | **X** | **X** | **X** | **X** |  | **X** | **X** |
| Gmm15 |  | **X** | **X** | **X** |  |  |  |  |
| Gmm22 | **X** | **X** | **X** | **X** | **X** | **X** | **X** | **X** |
| Gmm5 | **X** | **X** | **X** | **X** |  |  |  |  |
| GpB115 | **X** | **X** | **X** | **X** |  |  |  |  |
| GpB20b | **X** | **X** | **X** | **X** |  | **X** | **X** | **X** |
| GpC5b | **X** | **X** | **X** |  |  |  |  |  |
| Gmm9B |  | **X** |  |  |  |  |  |  |
| GmsCAG6 | **X** | **X** | **X** | **X** | **X** | **X** | **X** | **X** |
| GmcCA16c | **X** | **X** | **X** | **X** |  | **X** | **X** | **X** |
| GmsCAG2 | **X** | **X** | **X** | **X** | **X** | **X** | **X** | **X** |
| GmsCAG29B | **X** | **X** | **X** | **X** | **X** | **X** | **X** | **X** |
| GpCAG133 | **X** | **X** | **X** | **X** |  |  | **X** | **X** |
| Gff112 |  |  |  |  |  |  | **X** |  |
| Gpc101 |  | **X** | **X** | **X** | **X** | **X** | **X** | **X** |
| GpD18b |  | **X** | **X** | **X** |  |  |  |  |
| GpC10b | **X** | **X** | **X** | **X** | **X** |  | **X** | **X** |
| GpC26b | **X** | **X** | **X** | **X** |  |  |  |  |
| Gmm127 | **X** | **X** | **X** | **X** |  |  |  |  |
| GffC107 | **X** | **X** | **X** | **X** | **X** | **X** | **X** | **X** |
| GffD6 | **X** | **X** | **X** | **X** | **X** |  | **X** | **X** |
| GffD109 | **X** | **X** | **X** | **X** | **X** | **X** | **X** | **X** |

X: presence of the expected amplicon. In grey: markers selected for downstream genotyping purposes. One to three individuals per colony were used for the initial evaluation
