# Supplementary material for: Nuclear and Wolbachia-based multimarker approach for the rapid and accurate identification of tsetse species
Source: BMC Microbiol. 2018 Nov 23;18(Suppl 1):147. doi: 10.1186/s12866-018-1295-4 (PMC6251096; doi:10.1186/s12866-018-1295-4)
Supplement: Supplementary file 2 — ITS1 size variants as published in previous studies. (DOCX 17 kb) [file 12866_2018_1295_MOESM2_ESM.docx]

**Table S2: ITS1 size variants as published in previous studies**

| Taxon | ITS1 size variant | | | | | | | | | | | | Reference |
| --- | --- | --- | --- | --- | --- | --- | --- | --- | --- | --- | --- | --- | --- |
|  | Original primer pairs | | | | | | | Diagnostic PCR assays with modified primers | | | | |  |
|  | 880 | 778 | 919 | 633 | 597 | 618 | 543 | ~240 | ~240+  ~330 | 234+  239 | 234+  417 | 339 |  |
| *G. medicorum* | + |  |  |  |  |  |  |  |  |  |  |  | Dyer et al 2008 |
| *G. brevipalpis* |  | + |  |  |  |  |  |  |  |  |  |  |  |
| *G. pallidipes* |  |  | + |  |  |  |  |  |  |  |  |  |  |
| *G. austeni* |  |  |  | + |  |  |  |  |  |  |  |  |  |
| *G. tachinoides* |  |  |  |  | + |  |  |  |  |  |  |  |  |
| *G. f. quanzensis* |  |  |  |  |  | + |  |  |  |  |  |  |  |
| *G. f. fuscipes* |  |  |  |  |  | + |  |  |  |  |  |  |  |
| *G. p. gambiensis* |  |  |  |  |  |  | + |  |  |  |  |  |  |
| *G. p. palpalis* |  |  |  |  |  | + |  |  |  |  |  |  |  |
| *G. p. palpalis* |  |  |  |  |  |  |  | + | + |  |  |  | Dyer et al 2009 |
| *G. f. quanzensis* |  |  |  |  |  |  |  |  |  | + |  |  | Dyer et al 2011 |
| *G. f. martinii* |  |  |  |  |  |  |  |  |  |  | + |  |  |
| *G. f. fuscipes* |  |  |  |  |  |  |  |  |  |  |  | + |  |
