# Supplementary material for: Nuclear and Wolbachia-based multimarker approach for the rapid and accurate identification of tsetse species
Source: BMC Microbiol. 2018 Nov 23;18(Suppl 1):147. doi: 10.1186/s12866-018-1295-4 (PMC6251096; doi:10.1186/s12866-018-1295-4)
Supplement: Supplementary file 3 — Microsatellite markers’ cross species amplification in different Glossina taxa as referred in previous publications. (DOCX 24 kb) [file 12866_2018_1295_MOESM3_ESM.docx]

**Table S3: Microsatellite markers’ cross species amplification in different *Glossina* taxa as referred in previous publications.**

| SSR | Taxon | | | | | | | | | | | | | | Reference |
| --- | --- | --- | --- | --- | --- | --- | --- | --- | --- | --- | --- | --- | --- | --- | --- |
|  | pp | ff | tach | ms | mm | palli | swy | aus | brev | ongip | fuscipl | longi | mc | pg |  |
| *55.3* | 171-175 | 181-185 | - | - | - | nt | nt | nt | nt | nt | nt | nt | nt | nt | Solano et al. 1997 |
| *19.62* | 170-174 | 174-182 | - | - | - | nt | nt | nt | nt | nt | nt | nt | nt | nt |  |
| *69.22* | 194-200 | 192-192 | - | - | - | nt | nt | nt | nt | nt | nt | nt | nt | nt |  |
| *Gmm8* | + | + | + | nt | 125-131 | + | + | + | - | - | - | nt | nt | nt | Baker and Krasfur 2001 |
| ***Gmm14*** | **-** | **-** | **-** | **nt** | **153-211** | **+** | **-** | **-** | **-** | **-** | **-** | **nt** | **nt** | **nt** |  |
| *Gmm15* | - | - | - | nt | 185-195 | + | - | - | - | - | - | nt | nt | nt |  |
| *Gmm22* | - | - | - | nt | 133-145 | + | - | - | - | - | - | nt | nt | nt |  |
| *Gmm5B* | - | - | - | nt | 155-175 | - | - | - | - | - | - | nt | nt | nt |  |
| *Gmm9B* | - | - | - | nt | 140-180 | - | - | - | - | - | - | nt | nt | nt |  |
| *GmsCAG16* | - | - | - | nt | 120-140 | - | - | - | - | - | - | nt | nt | nt |  |
| *GmsCA16C* | + | + | + | nt | 200-210 | + | + | + | + | + | + | nt | nt | nt |  |
| *GmsCAG2* | + | + | - | nt | 130-145 | + | + | + | - | - | - | nt | nt | nt |  |
| *GmsCAG17B* | + | + | + | nt | + | + | + | + | - | - | - | nt | nt | nt |  |
| *GmsCAG29B* | - | - | - | nt | 175-190 | + | - | - | - | - | - | nt | nt | nt |  |
| *GpCAG133* | + | + | + | nt | 185-205 | + | + | + | - | - | - | nt | nt | nt |  |
| *Gmm127* | + | + | + | nt | 295-301 | + | + | - | - | - | - | nt | nt | nt |  |
| *Pgp1* | 124 | + | + | nt | nt | nt | nt | - | nt | nt | nt | nt | nt | nt | Luna et al 2001 |
| *Pgp8* | 192 | + | - | nt | nt | nt | nt | + | nt | nt | nt | nt | nt | nt |  |
| *Pgp11* | 178 | + | - | nt | nt | nt | nt | + | nt | nt | nt | nt | nt | nt |  |
| *Pgp13* | 201 | + | + | nt | nt | nt | nt | - | nt | nt | nt | nt | nt | nt |  |
| *Pgp17* | 191 | + | + | nt | nt | nt | nt | - | nt | nt | nt | nt | nt | nt |  |
| *Pgp20* | 194 | + | + | nt | nt | nt | nt | - | nt | nt | nt | nt | nt | nt |  |
| *Pgp22* | 279 | + | + | nt | nt | nt | nt | - | nt | nt | nt | nt | nt | nt |  |
| *Pgp24* | 215 | + | + | nt | nt | nt | nt | + | nt | nt | nt | nt | nt | nt |  |
| *Pgp28* | 103 | + | + | nt | nt | nt | nt | + | nt | nt | nt | nt | nt | nt |  |
| *Pgp29* | 237 | + | + | nt | nt | nt | nt | + | nt | nt | nt | nt | nt | nt |  |
| *Pgp33* | 208 | + | + | nt | nt | nt | nt | + | nt | nt | nt | nt | nt | nt |  |
| *Pgp34* | 364 | + | + | nt | nt | nt | nt | - | nt | nt | nt | nt | nt | nt |  |
| *Pgp35* | 202 | + | + | nt | nt | nt | nt | + | nt | nt | nt | nt | nt | nt |  |
| *Pgp38* | 225 | nt | nt | nt | nt | nt | nt | nt | nt | nt | nt | nt | nt | nt |  |
| *Pgp37* | 217 | nt | nt | nt | nt | nt | nt | nt | nt | nt | nt | nt | nt | nt |  |
| *GpA19a* | nt | + | nt | + | - | 142-189 | + | + | + | nt | nt | + | + | nt | Ouma et al 2003 |
| *GpA23b* | nt | + | nt | + | + | 172-215 | + | + | + | nt | nt | + | + | nt |  |
| *GpB6b* | nt | + | nt | + | - | 187-224 | + | + | + | nt | nt | + | + | nt |  |
| *GpB20b* | nt | + | nt | + | + | 139-200 | + | + | + | nt | nt | + | + | nt |  |
| *GpC5b* | nt | + | nt | + | + | 187-239 | + | + | + | nt | nt | + | + | nt |  |
| *GpC10b* | nt | + | nt | + | + | 283-314 | + | + | + | nt | nt | + | + | nt |  |
| *GpC26b* | nt | + | nt | + | + | 168-201 | + | + | + | nt | nt | + | + | nt |  |
| *GpD18b* | nt | + | nt | + | - | 220-229 | + | + | + | nt | nt | + | + | nt |  |
| *GpB115* | nt | - | nt | + | + | 133-177 | + | + | - | nt | nt | + | + | _ | Ouma et al 2006 |
| *GpC101* | nt | + | nt | + | + | 186-230 | + | + | + | nt | nt | + | + | + |  |
| *GpC107* | nt | + | nt | + | + | 202-217 | + | + | + | nt | nt | + | + | + |  |
| ***A10*** | **-** | **nt** | **nt** | **nt** | **nt** | **nt** | **nt** | **nt** | **nt** | **nt** | **nt** | **nt** | **nt** | **+** | Dyer et al 2008 |
| *Gff_B8* | nt | 183-217 | nt | nt | nt | nt | nt | nt | nt | nt | nt | nt | nt | nt | Brown et al 2008 |
| *Gff_C107* | nt | 189-245 | nt | nt | nt | nt | nt | nt | nt | nt | nt | nt | nt | nt |  |
| *Gff_D6* | nt | 259-279 | nt | nt | nt | nt | nt | nt | nt | nt | nt | nt | nt | nt |  |
| *Gff_D109* | nt | 153-177 | nt | nt | nt | nt | nt | nt | nt | nt | nt | nt | nt | nt |  |
| *Gff_A3* | nt | 227-258 | nt | nt | nt | nt | nt | nt | nt | nt | nt | nt | nt | nt |  |
| *Gff_A6* | nt | 257-267 | nt | nt | nt | nt | nt | nt | nt | nt | nt | nt | nt | nt |  |
| *Gff_A9* | nt | 170-174 | nt | nt | nt | nt | nt | nt | nt | nt | nt | nt | nt | nt |  |
| *Gff_A112* | nt | 121-133 | nt | nt | nt | nt | nt | nt | nt | nt | nt | nt | nt | nt |  |
| *Gff_B101* | nt | 268-308 | nt | nt | nt | nt | nt | nt | nt | nt | nt | nt | nt | nt |  |
| *Gff_A10* | nt | 184-213 | nt | nt | nt | nt | nt | nt | nt | nt | nt | nt | nt | nt |  |

Allele size or allele range is only given for the taxon where SSRs were originally developed.

+: presence of amplicon

-: absence of amplicon

nt: not tested

in bold: the two microsatellite markers selected to be included in the genotyping approach presented in this study
