# Supplementary material for: Nuclear and Wolbachia-based multimarker approach for the rapid and accurate identification of tsetse species
Source: BMC Microbiol. 2018 Nov 23;18(Suppl 1):147. doi: 10.1186/s12866-018-1295-4 (PMC6251096; doi:10.1186/s12866-018-1295-4)
Supplement: Supplementary file 4 — Wolbachia status in different Glossina taxa as referred in previous publications. (DOCX 14 kb) [file 12866_2018_1295_MOESM4_ESM.docx]

**Table S4: *Wolbachia* status in different *Glossina* taxa as referred in previous publications.**

| Taxon | *Wolbachia* | | Reference |
| --- | --- | --- | --- |
|  | Cytoplasmic | Chromosomal |  |
| *G. m. morsitans* | Low to fixed | Fixed | Doudoumis et al 2012 |
| *G. pallidipes* | low | Absent |  |
| *G. austeni* | Medium to fixed | Absent |  |
| *G. p. palpalis* | Absent | Absent |  |
| *G. p. gambiensis* | Absent to low | Absent |  |
| *G. brevipalpis* | Low to medium | Absent |  |
| *G. f. fuscipes* | Absent | Absent |  |
| *G. m. centralis* | Fixed (small sample) | Absent |  |
| *G. f. fuscipes* | Low to medium | not tested | Alam et al 2012 |
