# Supplementary material for: Nuclear and Wolbachia-based multimarker approach for the rapid and accurate identification of tsetse species
Source: BMC Microbiol. 2018 Nov 23;18(Suppl 1):147. doi: 10.1186/s12866-018-1295-4 (PMC6251096; doi:10.1186/s12866-018-1295-4)
Supplement: Supplementary file 5 — Abbreviations used in the Figures (taxon name and country of origin). (DOCX 15 kb) [file 12866_2018_1295_MOESM5_ESM.docx]

**List of abbreviations for figures**

| **Glossina taxa** | **Abbreviation** |  | **Country of origin** | **Abbreviation** |
| --- | --- | --- | --- | --- |
| Glossina austeni | Gaus |  | Angola | Ang |
| Glossina brevipalpis | Gbrev |  | Burkina Faso | BKF |
| Glossina fuscipes fuscipes | Gff |  | Central Africa Republic | CAR |
| Glossina fuscipes quanzensis | Gfq |  | Ethiopia | Eth |
| Glossina medicorum | Gmedi |  | Ghana | Ghan |
| Glossina morsitans morsitans | Gmm |  | Guinea | Guin |
| Glossina morsitans centralis | Gmc |  | Kenya | Ken |
| Glossina morsitans submorsitans | Gms |  | Mozambique | Moz |
| Glossina pallidipes | Gpal |  | Senegal | Sen |
| Glossina palpalis gambiensis | Gpg |  | Tanzania | Tanz |
| Glossina tachinoides | Gtach |  | Zambia | Zamb |
| Glossina swynnertoni | Gswyn |  | Zanzibar | Zanz |
|  |  |  | Zimbabwe | Zimb |
